# Hepatocyte Growth Factor Modulates MET Receptor Tyrosine Kinase and β-Catenin Functional Interactions to Enhance Synapse Formation

**DOI:** 10.1523/ENEURO.0074-16.2016

**Published:** 2016-08-29

**Authors:** Zhihui Xie, Kathie L. Eagleson, Hsiao-Huei Wu, Pat Levitt

**Affiliations:** 1 Department of Pediatrics, The Saban Research Institute, Children’s Hospital Los Angeles, Keck School of Medicine, University of Southern California, Los Angeles, California 90027; 2 Institute for the Developing Mind, The Saban Research Institute, Children’s Hospital Los Angeles, Keck School of Medicine, University of Southern California, Los Angeles, California 90027

**Keywords:** β-catenin, autism, MET receptor tyrosine kinase, synapse development

## Abstract

MET, a pleiotropic receptor tyrosine kinase implicated in autism risk, influences multiple neurodevelopmental processes. There is a knowledge gap, however, in the molecular mechanism through which MET mediates developmental events related to disorder risk. In the neocortex, MET is expressed transiently during periods of peak dendritic outgrowth and synaptogenesis, with expression enriched at developing synapses, consistent with demonstrated roles in dendritic morphogenesis, modulation of spine volume, and excitatory synapse development. In a recent coimmunoprecipitation/mass spectrometry screen, β-catenin was identified as part of the MET interactome in developing neocortical synaptosomes. Here, we investigated the influence of the MET/β-catenin complex in mouse neocortical synaptogenesis. Western blot analysis confirms that MET and β-catenin coimmunoprecipitate, but N-cadherin is not associated with the MET complex. Following stimulation with hepatocyte growth factor (HGF), β-catenin is phosphorylated at tyrosine^142^ (Y142) and dissociates from MET, accompanied by an increase in β-catenin/N-cadherin and MET/synapsin 1 protein complexes. In neocortical neurons *in vitro*, proximity ligation assays confirmed the close proximity of these proteins. Moreover, in neurons transfected with synaptophysin-GFP, HGF stimulation increases the density of synaptophysin/bassoon (a presynaptic marker) and synaptophysin/PSD-95 (a postsynaptic marker) clusters. Mutation of β-catenin at Y142 disrupts the dissociation of the MET/β-catenin complex and prevents the increase in clusters in response to HGF. The data demonstrate a new mechanism for the modulation of synapse formation, whereby MET activation induces an alignment of presynaptic and postsynaptic elements that are necessary for assembly and formation of functional synapses by subsets of neocortical neurons that express MET/β-catenin complex.

## Visual Abstract

**Figure:**
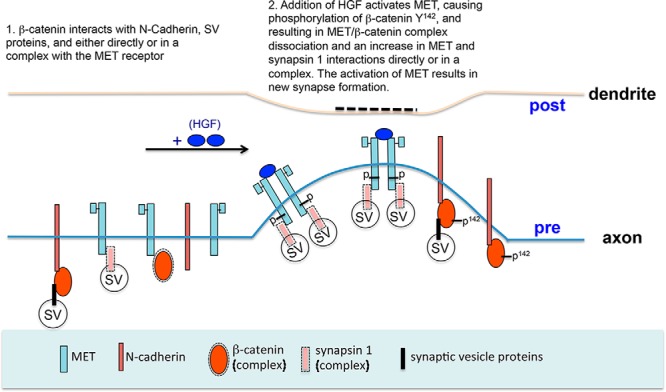

## Significance Statement

The gene encoding the MET receptor tyrosine kinase is associated with autism spectrum disorder, and influences typical and atypical synapse development and cortical circuit function. The present studies focus on determining potential molecular mechanisms through which the receptor functions in neocortical neurons during synaptogenesis. The findings show that the MET receptor interacts functionally with other proteins also implicated in promoting new synapse assembly, which is reduced upon disruption of the interactions. Thus, in some instances of autism spectrum disorder, disturbances of these molecular interactions may relate to the pathophysiology of cortical circuit development.

## Introduction

The MET receptor tyrosine kinase has been implicated in multiple neurodevelopmental processes (Peng et al., 2013), and thus outcomes from disruptions in MET function vary according to cell context. For example, in the forebrain, a risk allele for autism spectrum disorder (ASD) in the *MET* promoter, which reduces *MET* transcript and protein levels (Campbell et al., 2006, 2007), is correlated with altered circuit function in typical and ASD human populations (Rudie et al., 2012) and in gray matter growth (Hedrick et al., 2012). Further, following conditional deletion of *Met* in mice, there is an increase in local interlaminar drive onto layer V neurons in the neocortex and premature maturation of excitatory synapse function in the hippocampus (Qiu et al., 2011, 2014). Analyses *in vivo* and *in vitro* demonstrate that MET signaling modulates dendritic morphogenesis; spine volume; the clustering of postsynaptic proteins; excitatory synapse formation; and maturation in the neocortex, striatum, and hippocampus (Gutierrez et al., 2004; Tyndall and Walikonis, 2006; Nakano et al., 2007; Lim and Walikonis, 2008; Judson et al., 2010; Finsterwald and Martin, 2011; Qiu et al., 2011, 2014; Kawas et al., 2013; Eagleson et al., 2016; Peng et al., 2016). These developmental influences likely underlie the mature forebrain circuit phenotypes observed in the context of altered MET signaling. How MET receptor activation mediates these discrete cellular outcomes is only beginning to be addressed, with most focus on the diversity of downstream signaling pathways initiated following the activation of MET (Finsterwald and Martin, 2011; Eagleson et al., 2016). Evidence from cell lines, however, indicates that the repertoire of MET protein-interacting partners expressed by a cell also can modulate MET signaling to influence biological outcomes (Smyth and Brady, 2005; Wang et al., 2005; Zeng et al., 2006; Reshetnikova et al., 2007; DeAngelis et al., 2010; Bozkaya et al., 2012; Burghy et al., 2012; Lu et al., 2012; Niland et al., 2013). A recent coimmunoprecipitation (Co-IP)/mass spectrometry (MS) study identified the MET interactome with 72 proteins, including β-catenin, in isolated murine neocortical synaptosomes during the peak of synaptogenesis (Xie et al., 2016).

In the current study, we focused on the role of the MET/β-catenin protein complex in hepatocyte growth factor (HGF)-mediated neocortical synapse formation. Previous studies have shown the following: (1) that MET and β-catenin are expressed at the developing neocortical synapse (Phillips et al., 2001; Murase et al., 2002; Eagleson et al., 2013); (2) that MET activation increases synapse density on neocortical neurons *in vitro* (Eagleson et al., 2016); (3) that β-catenin regulates synaptic vesicle localization during presynaptic development in the hippocampus (Bamji et al., 2003; Yu and Malenka, 2003); and (4) that functional interactions between MET and β-catenin can be observed in hippocampal neurons, as well as cancer cell lines (Monga et al., 2002; Herynk et al., 2003; David et al., 2008), with the stability of the complex dependent upon the presence of HGF. MET and β-catenin physically interact with each other *in vitro*, and the activated MET receptor directly phosphorylates β-catenin at tyrosine^142^ (Y142; David et al., 2008). Consistently, following the addition of HGF in hippocampal neurons, β-catenin is phosphorylated at Y142 and dissociates from MET (Herynk et al., 2003; Rasola et al., 2007; David et al., 2008; Bhardwaj et al., 2013). Here, we used Co-IP/Western blot, proximity ligation assays (PLAs), and immunocytochemical analyses to determine how the MET/β-catenin complex might modulate neocortical synapse development in response to HGF. We report, following stimulation with HGF, a dynamic regulation of MET/β-catenin- and MET/synapsin 1-containing complexes in synaptosomes within minutes, a rapid increase in synapses in primary cultures of neocortical neurons. Both outcomes are dependent upon phosphorylation of β-catenin at Y142. We propose a model in which an axis of HGF/MET/β-catenin signaling modulates neocortical synapse development. Disruption of this signaling complex may contribute to ASD etiology.

## Materials and Methods

### Mice

Timed-pregnant C57BL/6^J^ mice were purchased from The Jackson Laboratory and the day of birth was considered postnatal day 0 (P0). Animals had free access to food and water and were housed in a 13/11 h light/dark cycle. All research procedures using mice were approved by the Institutional Animal Care and Use Committee at Children’s Hospital Los Angeles. All efforts were made to minimize animal suffering and to reduce the number of animals used.

### Plasmid construction

Mouse β-catenin full-length cDNA was cloned by PCR from an adult mouse brain cDNA library using high proof PfuUltra II Fusion HS DNA Polymerase (Agilent) according to the manufacturer protocol, using the following primer pair: 5' CTAGCTAGCTAGATGGATACGTATCGCTACATAATGGCTACTCAAGC 3' and 5' TGCTCTAGAGCATTACAGGTCAGTATCAAACCAGGCCAGCTGATT 3'. Purified β-catenin cDNA fragments were subcloned into a PCI expression vector (Promega) and transformed into DH5α competent cells (Invitrogen). PCI-β-catenin plasmids were purified using the Zyppy Plasmid Maxiprep Kit (Zymo Research) and PCR site-directed mutagenesis of β-catenin (β-catenin Y142F) performed according to a published strategy (Zheng et al., 2004) using the following primer pair: 5' GTTGTCAATTTGATTAACTTCCAGGATGACGCGGAACTTG 3' and 5' CAAGTTCCGCGTCATCCTGGAAGTTAATCAAATTGACAAC 3'. The β-catenin and β-catenin Y142F fragments were PCR amplified using the following primer pair: 5' CGGGATCCATGGATACGTATCGCTACATAATGGCTACTCAAGC 3' and 5' CGGGATCCTTACAGGTCAGTATCAAACCAGGCCAGCTGATT 3'.

The purified fragments were subcloned into a p3XFLAG-CMV-10 vector (Sigma-Aldrich) to generate p3XFLAG-CMV-10-β-catenin and p3XFLAG-CMV-10-β-cateninY142F plasmids. The fidelity of the entire coding sequences of all plasmids was confirmed by DNA sequencing (Genewiz).

### RNAscope

P14 mouse brains were fresh frozen in ice-cold isopentane and sectioned in the coronal plane at 25 μm. Sections were subjected to dual fluorescent *in situ* hybridization using the RNAscope Multiplex Fluorescent Reagent kit (Advanced Cell Diagnostics) according to manufacturer instructions. RNAscope probes and the regions used to generate the probes were as follows: *Met* (catalog #405301-C2, Advanced Cell Diagnostics; accession #NM_008591.2 region 3370-4286) and *β-catenin* (catalog #311741, Advanced Cell Diagnostics; accession #NM_007614.3 region 342-2511). Alexa Fluor 488 and Atto550 detection reagents were used to visualize *Met* and *β-catenin*, respectively. Images were acquired using a Zeiss LSM 710 confocal microscope with a 20× objective. The imaging parameters and *z*-axis were adjusted to bring the sample into focus. The parameters were maintained to capture focused optical images in each wavelength.

### Co-IP and Western blot analysis

All reagents for Co-IP and Western blots were from Sigma-Aldrich, unless otherwise noted. Crude synaptosomes were isolated from the neocortex of male and female P14 mice (Judson et al., 2010) were and resuspended in sodium bicarbonate-buffered oxygenated artificial CSF containing the following (in mM): NaCl 12.4, KCl 0.4, KH_2_PO_4_ 0.1 (Baker), CaCl_2_ 0.25 (Baker), MgCl_2_ 0.1, dextrose 1 (VWR). Either 25 ng/ml HGF (R&D Systems) or the same volume of vehicle (PBS) was added for 5 min (Co-IP experiments) or for 5, 10, and 20 min (β-catenin phosphorylation experiment) at 37^°^C to the synaptosomes. For Co-IP experiments, the synaptosomes were centrifuged at 16,000 × *g* for 15 min, and the pellets were lysed in Co-IP lysis buffer containing the following (in mM): HEPES 50, pH 7.4, EGTA 2, EDTA 2, NaF 30, sodium orthovanadate 10, β-glycerol phosphate 40, 1% Triton X-100, and a protease inhibitor cocktail. The lysate was centrifuged at 16,000 × *g* for 30 min, and the resulting supernatant used for Co-IP, with a goat anti-MET antibody (R&D Systems), a mouse anti-β-catenin antibody (BD Biosciences), or a rabbit anti-N-cadherin antibody (Santa Cruz Biotechnology). An equivalent amount of goat, mouse or rabbit IgG antibody (Jackson ImmunoResearch) was used in parallel lysates as a negative control. The Co-IP complexes were bound to protein G plus agarose beads (Pierce), after which the beads were washed in Co-IP lysis buffer plus 150 mM NaCl. The complexes were eluted from the beads by boiling in the final sample buffer (12.5 mM Tris-HCl, pH 6.8, 5% Glycerol, 0.4% SDS, 1% 2-mercaptoethanol, 0.02% bromophenol blue) and analyzed by Western blot. For the β-catenin phosphorylation experiment, the synaptosomes were centrifuged at 16,000 × *g* for 15 min followed by lysis in final sample buffer. Blots were probed with antibodies directed against β-catenin (1:2000; BD Biosciences), N-cadherin (1:500; Santa Cruz Biotechnology), synapsin1 (1:4000; EMD Millipore), synaptophysin1 (1:2000; EMD Millipore), phospho-MET (1:500; Cell Signaling Technology), and MET (1:500; Santa Cruz Biotechnology). Digital images of the Western blots were acquired using a CCD camera coupled to a UVP BioImaging System using VisionWorksLS Image Acquisition software (version 8.0, UVP).

### Semiquantification of the Co-IPs

**Figure 1.**
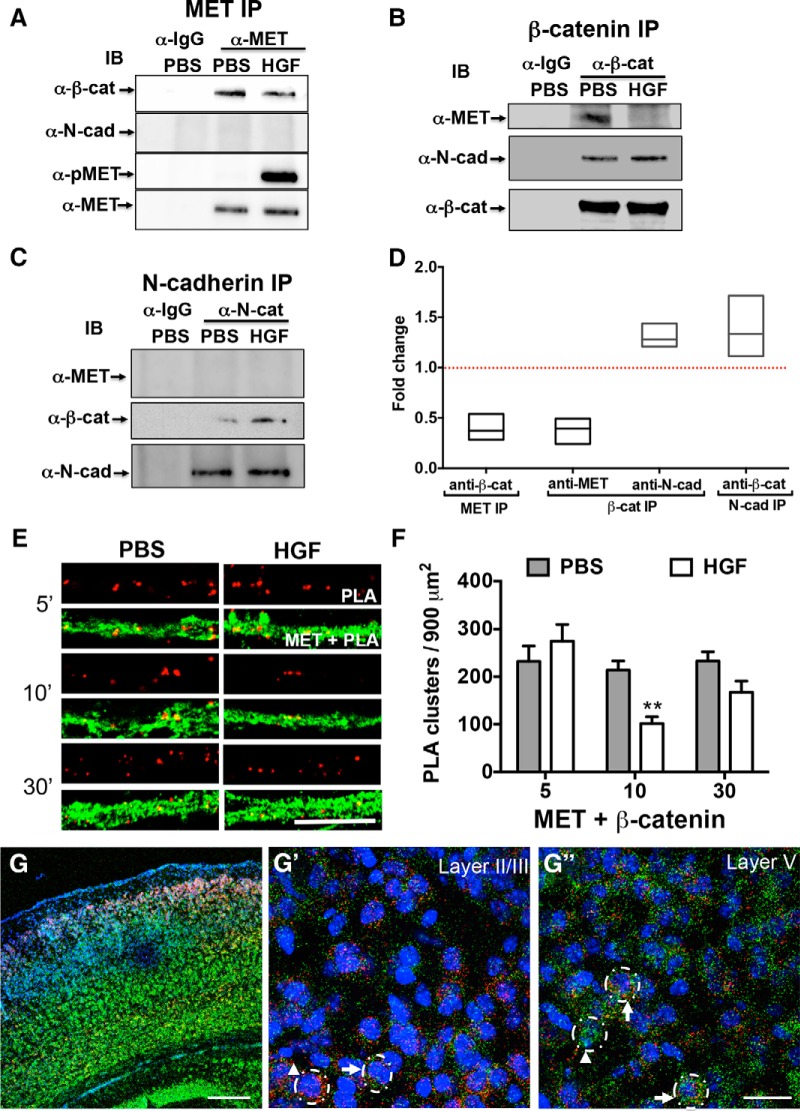
MET/β-catenin complexes during synapse development. ***A–C***, Representative Western blots of complexes immunoprecipitated from P14 cortical crude synaptosomes using anti-MET (***A***), anti-β-catenin (***B***), anti-*N*-cadherin (***C***), or control IgG antibody. This experiment was repeated three times using independent synaptosomal preparations. In PBS-treated synaptosomes, β-catenin (α-β-cat) and MET (α-MET) are detected in MET and β-catenin, but not in control IgG, pulldowns. Similarly, β-catenin and N-cadherin (α-N-cad) are evident in N-cadherin and β-catenin, but not in control IgG, pulldowns. MET is not detected in the N-cadherin pulldown. Stimulation of synaptosomes for 5 min with HGF results in reduced MET and β-catenin complexes, with a concomitant increase in the β-catenin and N-cadherin complexes (HGF lane vs PBS lane). An anti-phospo-MET antibody (α-pMET) was used to confirm HGF-induced activation of the MET receptor. ***D***, The fold change in the HGF-stimulated group compared with the PBS-stimulated group for each IP is presented as a box plot. The line bisecting the box represents the median. The horizontal red dash line indicates unchanged level (1.0) for comparison between HGF and PBS. *N* = 3 independent Co-IP experiments for each interaction. ***E***, Representative confocal microscopy images of PLA staining in primary cultures of neocortical neurons at 14 DIV following treatment with PBS or HGF for 5, 10, and 30 min. Red fluorescent profiles represent regions of PLA signal amplification denoting MET and β-catenin colocalization. For comparison, the total MET immunoreactivity (green fluorescence) in the same field is illustrated. Scale bar, 5 μm. ***F***, Quantitative analysis of the MET/β-catenin PLA signals. Error bars represent the SEM. *N* = 30 neurons from four independent culturing sessions in each group. **p < 0.01 (HGF vs PBS). ***G–G***″, Dual RNAscope *in situ* hybridization for *Met* (red) and *β-catenin* (green) in the P14 mouse cortex. Nuclei were labeled with DAPI (blue) to distinguish the cortical layers. Representative confocal microscopy images show *Met* expression in superficial and deep layers, with *β-catenin* expression across all layers, with more intense labeling in layers V and VI (***G***). Higher-magnification images from superficial (***G'***) and deep (***G″***) cortical layers. Dotted circles in ***G'*** and ***G″*** indicate RNAscope-labeled single cells. Arrows in ***G'*** and ***G″*** indicate *Met* and *β-catenin* colabeled cells. Arrowheads indicate *Met* (***G'***) or *β-catenin* (***G″***) single-labeled neurons. Scale bars: ***G***, 200 μm; ***G″*** (for ***G'*** and ***G″***), 25 μm.

Western blot analyses of Co-IPs in neocortical synaptosomes in the presence or absence of HGF were performed in three independent experiments. For each blot, representing an independent Co-IP experiment, the density of each immunoreactive band was measured in ImageJ (version 1.46r), and a background subtraction was applied. First, to account for different efficiencies of each pull down in each experiment, a ratio of coimmunoprecipitated protein (for example, Fig. 1*A*, β-catenin) to immunoprecipitated protein (for example, Fig. 1*A*, MET) was generated. Then, for each candidate, the data are expressed as the fold-change levels in the HGF-treated group compared with the PBS-treated group. The data are presented as box plots using GraphPad Prism 6.

### Primary neocortical neuron cultures

Primary cultures of neocortical neurons were prepared from P1 mice (Beaudoin et al., 2012) with the following minor modifications. In each culturing session, tissue from two male and female pups was pooled, and ∼50,000 cells/cm^2^ were seeded onto 12 mm coverslips (Carolina Biological Supply Company) in 24-well plates, which were precoated with poly-d-lysine (Sigma-Aldrich). Cells were initially plated in DMEM (Invitrogen) supplemented with 10% fetal bovine serum (FBS). After 4 h, the medium was replaced with neurobasal medium (Invitrogen) supplemented with B27 (Invitrogen) and l-glutamine (Invitrogen), and one-half of the volume of medium was replaced every 3 d. This condition results in slower growth than when using glial conditioned medium or a glial feeder layer. To achieve the sparse labeling of neurons, at 5 d *in vitro* (DIV), cultures were transfected with a p3XFLAG-CMV10-β-catenin or p3XFLAG-CMV10-β-catenin Y142F plasmid using a calcium phosphate transfection kit according to manufacturer instructions (Clontech). In some experiments, a synaptophysin-GFP (Syn-GFP) plasmid (obtained from L. Reichardt, University of California, San Francisco, San Francisco, CA) was cotransfected to label synaptic vesicles. At 14 DIV, 25 ng/ml HGF or the same volume of PBS was added to the medium for 5, 10, or 30 min (PLA assays) or for 10 min (Syn-GFP cluster assays). The experiments were repeated in at least three independent culturing sessions. At the end of the assay period, coverslips were fixed with 4% paraformaldehyde for 15 min at room temperature and processed for immunocytochemistry.

The PLA was used to determine spatial proximity between proteins that are immunolabeled with the Duolink *in situ* PLA kit (Sigma-Aldrich), as described previously (Eagleson et al., 2013). Immunofluorescent signals using dual imaging channels represent proteins that are within 40 nm or less of each other. The following antibody combinations with α-MET (1:50; R&D Systems) were used: (1) α-β-catenin (1:50; BD Biosciences); (2) α-synapsin1 (1:100; EMD Millipore); or (3) α-flag (1:200; Sigma-Aldrich). For the Syn-GFP cluster assays, coverslips were incubated with prechilled 100% methanol for 10 min at 4^°^C, then permeabilized with 0.1% Triton X-100 in PBS (PBST) for 20 min and blocked in blocking buffer (5% FBS in PBST) for 1 h at room temperature. Coverslips were incubated overnight at 4°C in the following primary antibody cocktails diluted in blocking buffer: (1) rabbit anti-bassoon (1:500; Cell Signaling Technology) and mouse anti-flag (1:1000; Sigma-Aldrich); or (2) mouse anti-PSD-95 (1:1000; Thermo Scientific) and rabbit anti-flag (1:1000). Following three washes with PBS, coverslips were incubated for 1 h at room temperature in the following cocktails of Alexa Fluor-labeled secondary antibodies (all at 1:1000; Life Technologies): (1) 546 goat-anti-mouse and 633 goat-anti-rabbit for bassoon/flag staining; or (2) 546 goat anti-rabbit and 633 goat-anti-mouse for PSD-95/flag staining. After three washes in PBS, coverslips were mounted onto glass slides with Prolong Mounting Medium (Life Technologies).

### Image analysis

Images were captured using an automated laser-scanning confocal microscope (LSM 710, Zeiss) with a 60× oil-immersion objective. The focal point of the beam and the *z*-axis were adjusted until an appropriate focus was reached. All images in a given culturing session were captured and analyzed with the same exposure time and settings. Note that the visualization of spines requires a longer exposure than that needed to visualize the labeling of synaptic proteins in the linear range. Because the settings were optimized for the analysis of synapses, there is an apparent absence of dendritic protrusions in the captured images. For PLA analyses, in each culturing session, six to eight fields were randomly imaged for each treatment group. Quantitative measures of MET association with select protein partners were obtained by counts of PLA clusters/area of dendrite using published methods (Eagleson et al., 2013). For the Syn-GFP cluster assays, axons from eight transfected neurons, based on Syn-GFP labeling, were imaged for each treatment group in each culturing session. Images were imported into ImageJ (NIH) for analysis. Syn-GFP puncta colabeled with bassoon or PSD-95, and single-labeled Syn-GFP puncta, were identified using an automated plugin to ImageJ (https://github.com/Pat-Levitt/SynapseCounter; Wang et al., 2015), with thresholds set in each channel independently. Once the threshold was set for a given culturing session, the same threshold was used throughout the analyses. The following parameters were then measured for each colabeled Syn-GFP^+^ puncta using the “analyze particles” tool, as follows: (1) density (average number of puncta per 100 μm of axon length); (2) integrated density (the product of the puncta area and the average gray value within that area); (3) major length (the length of the major axis of Syn-GFP fluorescence signal expressed as the average Feret’s diameter); and (4) the size of the puncta.

### Statistics

Data were expressed as the mean ± SEM. For each experimental manipulation, data were collected from at least three independent culturing sessions. Individual neurons were considered as samples (Sun and Bamji, 2011; Crowell et al., 2015), and sample size varied between studies and is indicated in the figure legends. The normality of the data was tested using the D’Agostino and Pearson omnibus normality test (D'Agostino and Pearson, 1986). If the data were not normally distributed, either the data were transformed to meet the assumption of normal distribution or a nonparametric test was applied. Specifically, the data in Figures 1*F*, 4*D*, and 6*C* were transformed with a square root, and the data in Figure 6*D* were transformed with a natural logarithm to meet the assumption of normality for two-way ANOVA analyses. For two-way ANOVAs, means were compared to determine the effects of treatment (PBS and HGF) × treatment time, or treatment × transfected plasmid (wild-type β-catenin and β-catenin-Y142F), and the interaction between those factors. If a significant effect was detected, a Bonferroni’s multiple-comparisons test was performed to determine the possible source of interactions. When the data were not normally distributed, the Mann–Whitney *U* test was used to compare differences between PBS- and HGF-treated groups. The unpaired *t* test with Welch’s correction was applied to compare differences between PBS- and HGF-treated groups if the data were normally distributed. For all tests, *p* values are reported to the fourth decimal place, and values <0.05 are considered to be significant. Statistical analyses and the preparation of graphs were performed using GraphPad Prism 6.0.

## Results

### HGF downregulates the MET and β-catenin complex during synapse development

Previously, a discovery-based Co-IP/MS method was used to detect the MET interactome in neocortical synaptosomes at the peak of synaptogenesis (P14) and identified β-catenin as a member of the MET interactome (Xie et al., 2016). In this study, we took several approaches to validate and characterize the functional relevance of the MET/β-catenin complex. First, we confirmed the presence of a MET/β-catenin complex in neocortical crude synaptosomes using Western blot analysis of Co-IPs. Specifically, β-catenin was detected in MET-immunoprecipitated complexes (Fig. 1*A*) and, conversely, MET was present in β-catenin-immunoprecipitated complexes (Fig. 1*B*). Neither protein was detected in complexes that had been immunoprecipitated with control IgG. Activation of the MET receptor following treatment of the synaptosomes with HGF for 5 min was accompanied by a significant decrease in MET/β-catenin complex [mean fold change (HGF/PBS), 0.3987 (95% CI, 0.0773–0.7201) for β-catenin in MET IP; mean fold change (HGF/PBS), 0.3760 (95% CI 0.0606–0.6915) for MET in β-catenin IP; Fig. 1*D*]. The β-catenin and N-cadherin complex also occurs during synapse formation (Uchida et al., 1996). Interestingly, while we confirmed this complex following immunoprecipitation with β-catenin and N-cadherin antibodies (Fig. 1*B*,*C*), N-cadherin could not be detected in MET pulldowns (Fig. 1*A*). These data show that β-catenin forms separate complexes with MET and with N-cadherin. Moreover, following HGF stimulation, there is an increase in N-cadherin/β-catenin complex [mean fold change (HGF/PBS), 1.309 (95% CI 1.018–1.600) for N-cadherin in β-catenin IP; mean fold change (HGF/PBS), 1.388 (95% CI 0.6342–2.141) for β-catenin in N-cadherin IP; Fig. 1*D*] that complement the reduced MET/β-catenin complex (Fig. 1*A*,*B*,*D*).

We next used the PLA to examine the proximity of MET/β-catenin in primary cultures of neocortical neurons. At 14 DIV, PLA signal was detected in the presence of MET and β-catenin antibodies (Fig. 1*E*), indicating a close proximity between these two proteins. There was a significant effect of treatment (PBS vs HGF) on the magnitude of the PLA signal (*F*_(1,174)_ = 9.401, *p* = 0.0025, Fig. 1*F*). Pairwise analysis revealed that there was no effect for HGF stimulation for 5 min (PBS vs HGF: *p* ^a^ = 0.8383, df = 174; Table 1), but there was a significant reduction in PLA signal after HGF stimulation for 10 min (*p* ^b^ = 0.0002, df = 174). Specifically, after stimulation for 10 min, there was an ∼50% decrease in the density of PLA clusters in HGF-treated compared with PBS-treated cultures (Fig. 1*F*). The PLA signal returned to prestimulation levels 30 min following HGF treatment (PBS vs HGF: *p* ^c^ = 0.0723, df = 174; Fig. 1*F*). Together, the Co-IP/Western and PLA analyses demonstrate that MET/β-catenin proximity is regulated in an HGF-dependent manner.

**Table 1:** Statistical table

Figures	Comparisons	Label	Data structure	Type of test	Power
1F	PBS vs HGF (5 min)	a	Normal distribution after transformation	Two-way ANOVA with Bonferroni's multiple comparisons test	0.26
	PBS vs HGF (10 min)	b			1.00
	PBS vs HGF (30 min)	c			0.93
2C	PBS vs HGF	d	Non-normal distribution	Mann–Whitney test	0.99
3C	PBS vs HGF	e	Normal distribution	Unpaired *t* test with Welch's correction	1.00
4D	β-catenin vs β-cateninY142F PBS treatment)	f	Normal distribution after transformation	Two-way ANOVA Bonferroni’s multiple comparisons test	0.17
	β-catenin vs β-cateninY142F HGF treatment)	g			1.00
	PBS vs HGF (β-catenin transfection)	h			1.00
	PBS vs HGF (β-cateninY142F transfection)	i			0.01
5E	PBS vs HGF (Syn-GFP/bassoon)	j	Non-normal distribution	Mann–Whitney test	0.99
	PBS vs HGF (Syn-GFP/PSD-95)	k	Non-normal distribution	Mann–Whitney test	0.99
	PBS vs HGF (Syn-GFP)	l	Non-normal distribution	Mann–Whitney test	0.94
5F	PBS vs HGF (Syn-GFP/bassoon)	m	Non-normal distribution	Mann–Whitney test	0.94
	PBS vs HGF (Syn-GFP/PSD-95)	n	Non-normal distribution	Mann–Whitney test	0.78
	PBS vs HGF (Syn-GFP)	o	Non-normal distribution	Mann–Whitney test	0.82
5G	PBS vs HGF (Syn-GFP/bassoon)	p	Non-normal distribution	Mann–Whitney test	0.90
	PBS vs HGF (Syn-GFP/PSD-95)	q	Non-normal distribution	Mann–Whitney test	0.75
	PBS vs HGF (Syn-GFP)	r	Normal distribution	Unpaired *t* test with Welch's correction	0.92
5H	PBS vs HGF (Syn-GFP/bassoon)	s	Non-normal distribution	Mann–Whitney test	0.91
	PBS vs HGF (Syn-GFP/PSD-95)	t	Non-normal distribution	Mann–Whitney test	0.90
	PBS vs HGF (Syn-GFP)	u	Non-normal distribution	Mann–Whitney test	0.88
6C	β-catenin vs β-cateninY142F PBS treatment, Syn-GFP/bassoon)	v	Normal distribution after transformation	Two-way ANOVA Bonferroni's multiple comparisons test	0.00
	PBS vs HGF (β-catenin transfection, Syn-GFP/bassoon)	w			0.98
	PBS vs HGF (β-cateninY142F transfection, Syn-GFP/bassoon)	x			0.69
6D	β-catenin vs β-catenin Y142F PBS treatment, Syn-GFP/PSD-95)	y	Normal distribution after transformation	Two-way ANOVA Bonferroni's multiple comparisons test	0.00
	PBS vs HGF (β-catenin transfection, Syn-GFP/PSD-95)	z			1.00
	PBS vs HGF (β-cateninY142F transfection, Syn-GFP/PSD-95)	aa			0.00

To meet the assumption of normality, the data from Figures 1F, 4D, and 6C were transformed with the square root, and the data from Figure 6D were transformed with the natural logarithm. The normality of the data was tested using the D’Agostino and Pearson omnibus normality test.

In the third set of experiments, we used RNAscope to examine the coexpression of *Met* and *β-catenin* in the mouse neocortex at P14, providing an anatomical context for MET and β-catenin complex *in vivo*. There was a gradient of *Met* and *β-catenin* coexpression across neocortical layers (Fig. 1*G*). Specifically, in layers II/III, there were many neurons that coexpressed the *Met* and *β-catenin* transcripts (Fig. 1*G*' ). In deeper layers, the signal intensity of puncta was greater in colabeled neurons (Fig. 1*G*″ ). In both superficial and deep layers, there also were single-labeled *Met* or *β-catenin* pyramidal neurons (Fig. 1*G'*,*G″* ). These data indicate that the MET/β-catenin complex resides in subsets of neocortical neurons during the peak period of synapse formation. This heterogeneity in neuronal coexpression may account in part for the disruption of physiological functions in a subset of neocortical synapses after *Met* deletion (Qiu et al., 2011).

### HGF increases the MET/synapsin 1 complex during synapse development

**Figure 2.**
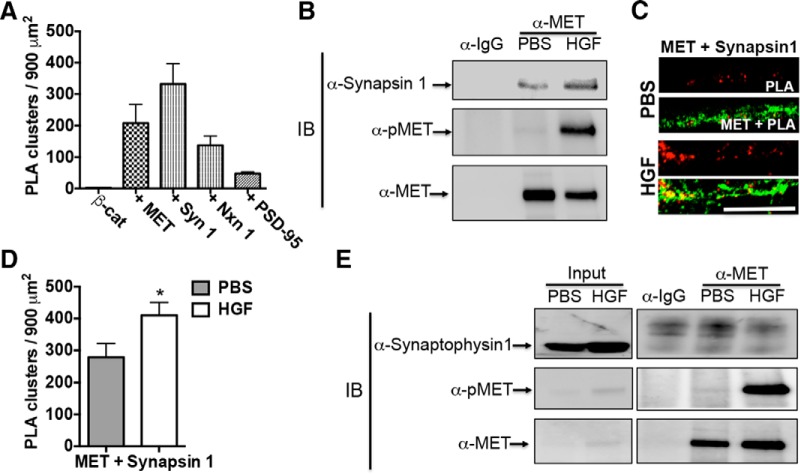
HGF recruits additional synapsin 1 to MET receptor complex. ***A***, Quantitative analysis of PLA signals generated with β-catenin alone (β-cat), β-catenin with MET (+ MET), β-catenin with synapsin 1 (+ Syn 1), β-catenin with Neurexin 1 (+ Nxn 1), and β-catenin with PSD-95 (+ PSD-95). Error bars represent the SEM; *N* = 6–8 cells from independent cultures for each group. ***B***, Representative Western blots of complexes immunoprecipitated from P14 cortical crude synaptosomes using an anti-MET or control IgG antibody. This experiment was repeated three times using independent synaptosomal preparations. In PBS-treated synaptomsomes, synapsin 1 and MET are detected in the MET, but not IgG, pulldowns. Stimulation of the synaptosomes for 5 min with HGF increased the MET/synapsin 1 complex (HGF vs PBS lane). An anti-phospo-MET antibody (α-pMET) was used to confirm HGF-induced activation of the MET receptor. ***C***, Representative confocal microscopy images of PLA staining in primary cultures of neocortical neurons at 14 DIV following treatment with PBS or HGF for 10 min. Red fluorescent profiles represent regions of PLA signal amplification denoting MET and synapsin 1 colocalization. For comparison, the total MET immunoreactivity (green fluorescence) in the same field is illustrated. Scale bar, ***C*** (for ***B***, ***C***), 5 μm. ***D***, Quantitative analysis of the MET/synapsin 1 PLA signals. Error bars represent the SEM; *N* = 30 cells from five independent cultures in each group. *p < 0.05 (HGF vs PBS). ***E***, Representative Western blots of complexes that were immunoprecipitated from P14 cortical crude synaptosomes using an anti-MET or control IgG antibody. MET is detected in the MET, but not IgG, pulldowns. Stimulation of the synaptosomes for 5 min with HGF results in phospho-MET detection in MET pulldowns (HGF vs PBS lane). A single synaptophysin 1 band is readily detected in the input sample prior to IP. In contrast, the postimmunoprecipitation sample has only nonspecific bands in all Co-IP groups, indicating that synaptophysin 1 does not coimmunoprecipitate with MET under these conditions.

Following HGF stimulation, β-catenin dissociates from the MET complex. We hypothesized that the activation of MET could result in the recruitment of other protein partners into the receptor complex. MET is localized in presynaptic and postsynaptic compartments, but with predominant enrichment in presynaptic compartments in the developing neocortex (Eagleson et al., 2013) and cultured neocortical neurons. β-Catenin also is densely colocalized with presynaptic markers synapsin 1 and neurexin 1 compared with postsynaptic marker PSD-95 in cultured neocortical neurons (Fig. 2*A*). Thus, the experiments here focused on the impact of MET receptor activation on presynaptic proteins. In P14 neocortical synaptosomes, synapsin 1 is coimmunoprecipitated with MET (Fig. 2*B*). Further, in contrast to β-catenin, following the activation of MET by HGF stimulation for 5 min, additional synapsin 1 was recruited to MET complexes [mean fold change (HGF/PBS), 1.804; 95% CI 1.114–2.494; Fig. 2*B*]. Consistent with this, the density of the PLA signal generated by MET and synapsin 1 antibody labeling in neocortical neurons *in vitro* was significantly increased (∼1.5 fold, *p* ^d^ = 0.0109, df = 58) following HGF addition, compared with PBS treatment (Fig. 2*C*,*D*). In contrast with the MET/synapsin 1 complex, MET and synaptophysin 1 do not coimmunoprecipitate under conditions with or without HGF stimulation (Fig. 2*E* ). These data suggest that there is an increase in a functional MET/synapsin 1 complex following HGF stimulation.

### HGF regulates MET and β-catenin complex through phosphorylation of β-catenin at Y142

**Figure 3.**
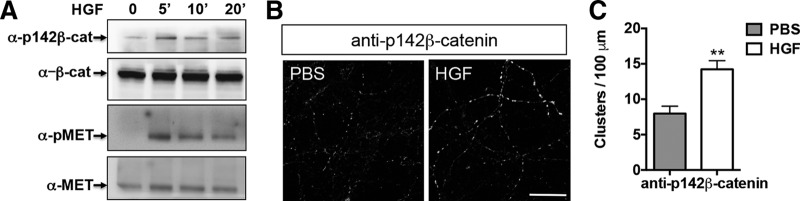
HGF promotes phosphorylation of β-catenin at Y142. ***A***, Representative Western blots of crude neocortical synaptosomes following stimulation with HGF stimulation for 0, 5, 10, and 20 min. This experiment was repeated three times using independent synaptosomal preparations. The level of β-catenin phosphorylated at Y142 (p142-β-cat) increased in the presence of HGF, peaking at 5 min. Note the expected increase, followed by a time-dependent decrease, in phospho-MET (pMET) levels in response to HGF. Total levels of β-catenin (β-cat) and MET are unchanged. ***B***, Representative confocal microscopy images of primary neocortical neurons at 14 DIV following stimulation for 5 min with PBS or HGF (25 ng/ml). This experiment was repeated in two independent culturing sessions. Note the increase in immunostaining of p142- β-catenin in the presence of HGF. Scale bar, 20 μm. ***C***, Quantitative analysis of the p142- β-catenin clusters. Error bars represent the SEM; *N* = 26 cells from two independent cultures in each group. *p < 0.05 (HGF vs PBS).

Previous reports demonstrated phosphorylation of β-catenin at Y142 in response to HGF in cancer cells and cultured hippocampal neurons (Herynk et al., 2003; Rasola et al., 2007; David et al., 2008; Bhardwaj et al., 2013). Similarly, we found that, following the addition of HGF to P14 neocortical synaptosomes, the level of phosphorylated of β-catenin (Y142) was increased, reaching a peak after 5 min, and declining toward prestimulation levels by 20 min (Fig. 3*A*). Moreover, in cultured neocortical neurons at 14 DIV, the number of immunoreactive puncta, labeled with an antibody that recognizes Y142-phosphorylated β-catenin (Strom et al., 2007; David et al., 2008), significantly increased after HGF treatment for 10 min (approximately twofold; *p* ^e^ = 0.003, *t* = 3.882, df = 51; Fig. 3*B*,*C*). Together, these results indicate that HGF can regulate β-catenin phosphorylation at Y142 in the neocortex during the period of synapse formation.

**Figure 4.**
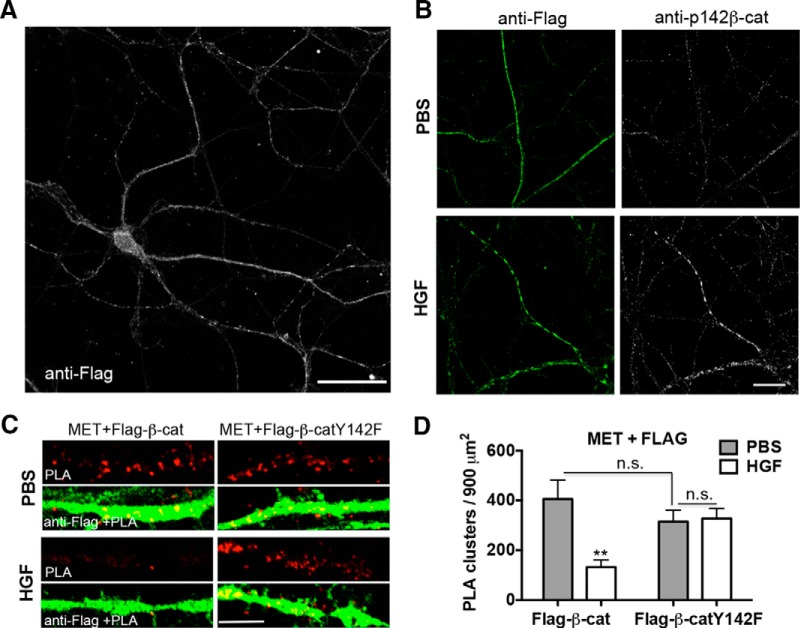
HGF modulates MET/β-catenin complex via phosphorylation of β-catenin at Y142. ***A***, Neurons were transfected with flag-tagged wild-type β-catenin (Flag-β-cat). Representative confocal microscopy image of transfected neuron with total flag immunoreactivity (white) was shown. Note that transfected β-catenin was distributed along the entire neuron and processes. Scale bar, 50 μm. ***B***, Representative confocal microscopy images of Flag-β-cat-transfected neurons with total flag (green) and p142- β-catenin immunoreactivity (white). Note the positive immunostaining of p142- β-catenin in the Flag-β-cat-transfected neuron with stimulation of HGF. Scale bar, 25 μm. ***C***, Representative confocal microscopy images of PLA staining of MET and flag in primary cultures of neocortical neurons at 14 DIV following treatment with PBS or HGF for 10 min. Neurons were transfected with Flag-β-cat or β-cateninY142F (Flag-β-catY142F) at 5 DIV. Red fluorescent profiles represent regions of PLA signal amplification denoting MET and flag colocalization. For comparison, the total flag immunoreactivity (green fluorescence) in the same field is illustrated. Scale bar, 5 μm. ***D***, Quantitative analysis of the MET/flag PLA signals. Error bars represent the SEM; *N* = 22 cells from three independent cultures in each group. Note that there is a decrease in PLA signal with HGF in the wild-type Flag-β-catY, but that there is no change with HGF in the Flag-β-catY142F condition. **p < 0.01 (HGF vs PBS in Flag-β-cat-transfected group); *p* = n.s. (Flag-β-cat vs Flag-β-catY142F with PBS stimulation, HGF vs PBS in Flag-β-catY142F-transfected group).

To address the possibility that phosphorylation of β-catenin at Y142 modulates the extent to which MET and β-catenin functionally interact, neocortical neurons were transfected with wild-type β-catenin that can be phosphorylated at Y142 with HGF treatment (Fig. 4*A*,*B*) or a mutant form of β-catenin that cannot be phosphorylated at Y142 (β-cateninY142F; David et al., 2008). PLA analyses at 14 DIV demonstrated that, consistent with our previous data with endogenous β-catenin and MET associated, either directly or indirectly, with transfected β-catenin and MET, this association is downregulated by HGF (Fig. 4*C*). Quantitative analysis revealed a significant functional association between the construct transfected (wild-type β-catenin vs β-cateninY142F) and treatment (PBS vs HGF) on the density of PLA clusters (*F*_(1,84)_ = 9.570, *p* = 0.0027; Fig. 4*C*,*D*). Pairwise analysis revealed that this effect was due to differences in the PLA signal following HGF stimulation. Specifically, there was no significant difference in the density of PLA clusters between wild-type β-catenin and β-cateninY142F in the absence of HGF (*p* ^f^ = 0.8269, df = 84; Fig. 4*C*,*D*), demonstrating that β-cateninY142F still associated in a complex with MET. Following stimulation with HGF, however, there was a significant difference between wild-type β-catenin and β-cateninY142F (*p* ^g^ = 0.0013, df = 84; Fig. 4*C*,*D*). Specifically, there was an ∼70% decrease in the density of PLA clusters in neurons transfected with wild-type β-catenin (*p* ^h^ = 0.0002, df = 84), but no significant difference in PLA signal in flag-β-cateninY142F-transfected neurons (*p* ^i^ > 0.9999, df = 84; Fig. 4*C*,*D*), compared with PBS. These data demonstrate that HGF-stimulated phosphorylation of β-catenin at Y142 is required for the subsequent dissociation of the MET/β-catenin complex. We noted that the transfected β-catenin or β-cateninY142F was distributed in the entire neuron (Fig. 4*A*), but the PLA signals are rarely present in dendrites. The labeling pattern may suggest that HGF stimulation impacts the functional association of MET with transfected β-catenin at presynaptic sites, but we cannot exclude postsynaptic complex interactions.

### HGF increases the density of Syn-GFP/bassoon and Syn-GFP/PSD-95 clusters in neocortical neurons

**Figure 5.**
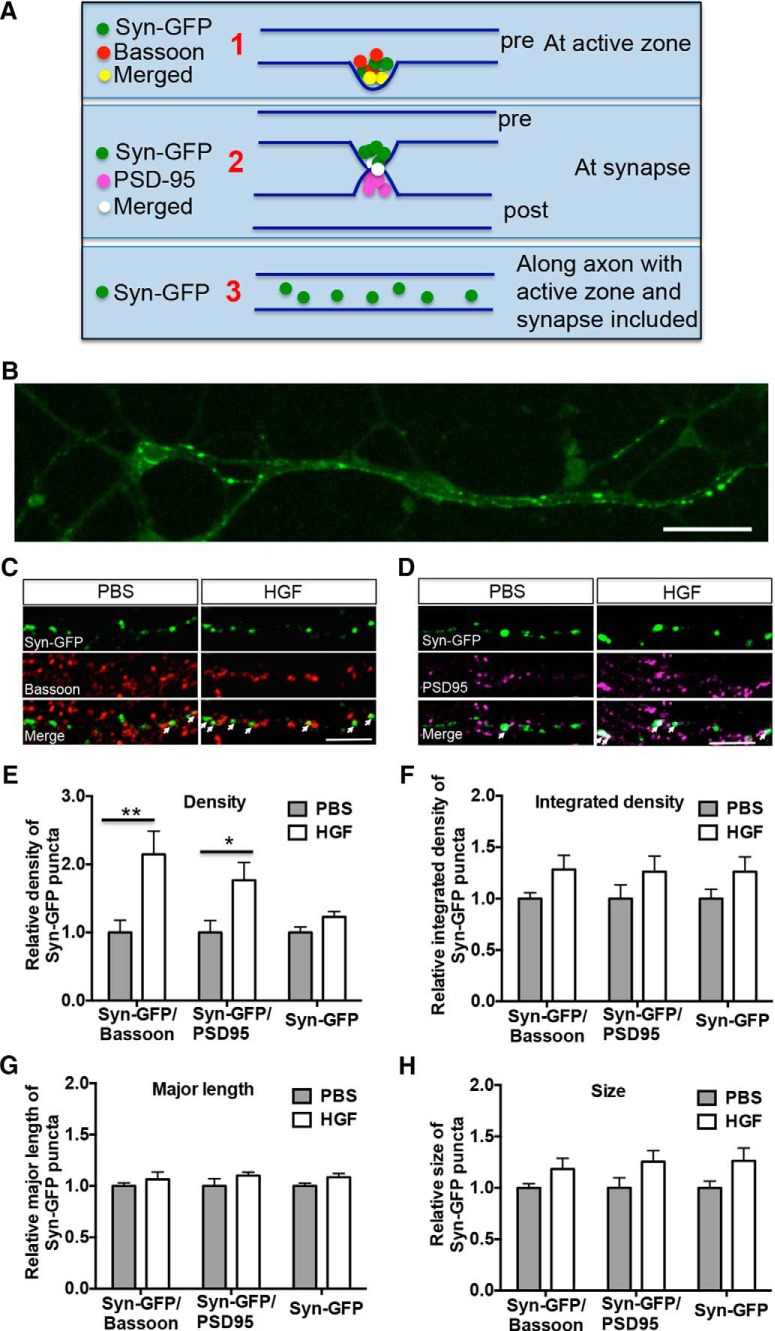
MET activation increases the density of Syn-GFP/bassoon and Syn-GFP/PSD-95 clusters in neocortical neurons. ***A***, Diagram of Syn-GFP cluster assay. Neocortical neurons were transfected with Syn-GFP at 5 DIV and treated with PBS or HGF (25 ng/ml) for 10 min at 14 DIV. Measurements were made of (1) Syn-GFP/bassoon (a marker of the active zone) colabeled clusters, (2) Syn-GFP/PSD-95 (a marker of the postsynaptic density) colabeled clusters, and (3) clusters labeled with Syn-GFP along the axon (with active zone and synapse included). ***B***, Representative confocal microscopy image of Syn-GFP-transfected (green) neurons. Note that Syn-GFP clusters are present along axons but not dendrites. Scale bar, 50 μm. ***C***, ***D***, Representative confocal microscopy images of Syn-GFP (green), bassoon (red), and PSD-95 (magenta) immunoreactivity in neocortical neurons. White arrows indicate clusters colabeled with Syn-GFP/bassoon (***C***) or Syn-GFP/PSD-95 (***D***). Scale bars: ***C***, ***D***, 5 μm. ***E–H***, Quantitative analysis of the density (***E***), integrated density (***F***), major length (***G***), and size (***H***) of Syn-GFP clusters. Each parameter was normalized in each culturing session to the mean value of Syn-GFP clusters in the PBS-treated group. Error bars represent the SEM; *N* = 18 cells from three independent cultures in each group. Increases in colabeling of presynaptic/postsynaptic markers are evident following HGF stimulation. *p < 0.05, **p < 0.01 (HGF vs PBS).

Given the increased MET/synapsin 1 complex following activation of the receptor, we reasoned that HGF/MET signaling might contribute to presynaptic development through an alteration in protein complexes. To address this, we transfected neurons with Syn-GFP, which is a marker of synaptic vesicles, localized at presynaptic sites and axon but not at dendrites (Fig. 5*B*; Bamji et al., 2003). MET/synaptophysin are not found in the same protein complex. Following transfection, neurons were treated with HGF for 10 min at 14 DIV. At the end of the treatment period, we categorized Syn-GFP puncta (Fig. 5*A*) according to whether they were (1) colabeled with bassoon (Fig. 5*C*), an active zone marker; (2) colabeled with PSD-95 (Fig. 5*D*), a postsynaptic marker; or (3) total labeled Syn-GFP puncta (Syn-GFP), including puncta colabeled with bassoon or PSD-95 and puncta labeled with Syn-GFP alone. Mann–Whitney statistical analyses revealed a significant increase in the density of Syn-GFP puncta colabeled with bassoon (∼2.1-fold, *U*_(34)_ = 80, *p* ^j^ = 0.0087; Fig. 5*C*,*E*) or with PSD-95 (∼1.8-fold, *U*_(34)_ = 84, *p* ^k^ = 0.0129, Fig. 5*D*,*E*) in HGF-treated compared with PBS-treated cultures. In contrast, there was no significant difference in the density of clusters labeled with Syn-GFP (*U*_(34)_ = 103, *p* ^l^ = 0.0633). We further characterized Syn-GFP puncta colabeled with bassoon or PSD-95, measuring parameters previously shown to be influenced by β-catenin (Sun et al., 2009). There was no significant effect of HGF treatment on the integrated density (Fig. 5*F*), major length (Fig. 5*G*), or size (Fig. 5*H*) of Syn-GFP puncta, which approximate the size of the synaptic vesicle pool (Sun et al., 2009). Together, these data suggest that HGF promotes the rapid assembly of synaptic vesicles at active zones to increase the formation of nascent synapses, but does not further cause the accumulation of synaptic vesicles at existing synaptic sites.

### Phosphorylation of β-catenin at Y142 is required for the HGF-induced increase in the density of Syn-GFP/bassoon and Syn-GFP/PSD-95 clusters

**Figure 6.**
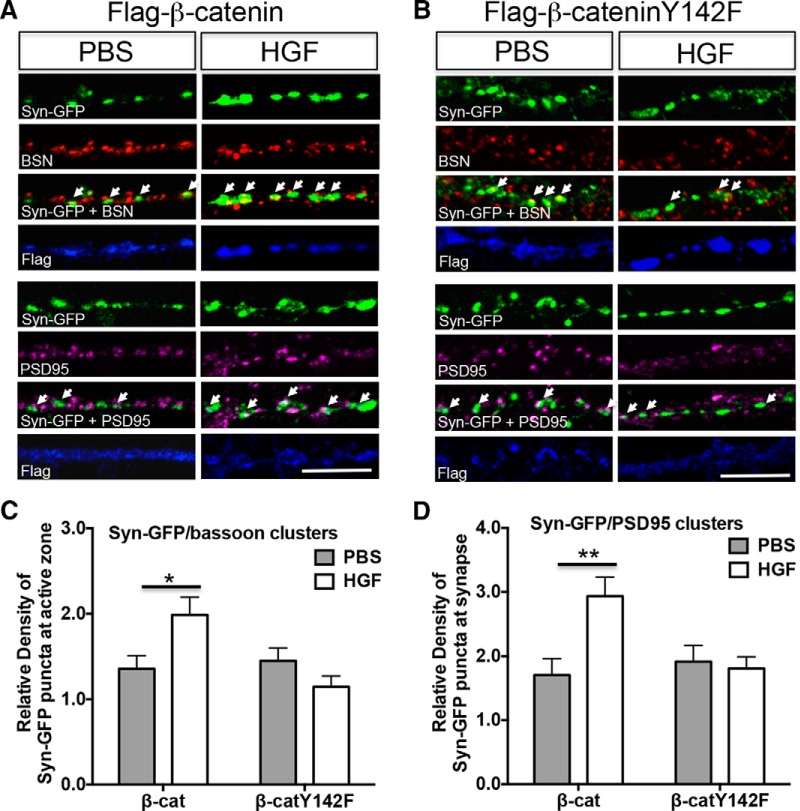
Phosphorylation of β-catenin at Y142 is required for the HGF-induced increase in Syn-GFP/bassoon and Syn-GFP/PSD-95 clusters. ***A***, ***B***, Representative confocal microscopy images of Syn-GFP (green), bassoon (red), PSD-95 (magenta), and flag (blue) immunoreactivity in neocortical neurons at 14 DIV after treatment with PBS or HGF (25 ng/ml) for 10 min. The neurons were cotransfected with Syn-GFP and either flag-β-catenin (***A***) or flag-β-cateninY142F (***B***) plasmids at 5 DIV. White arrows indicate clusters colabeled with Syn-GFP/bassoon (top panels) or Syn-GFP/PSD-95 (bottom panels). Scale bars: ***A***, ***B***, 5 μm. ***C***, ***D***, Quantitative analysis of the density of Syn-GFP/bassoon (***C***) and Syn-GFP/PSD-95-colabeled clusters in β-catenin (β-cat)- or β-cateninY142F (β-catY142F)-transfected neurons. Each parameter was normalized in each culturing session to the mean value of Syn-GFP clusters in the PBS-treated group. Error bars represent the SEM; *N* = 24 cells from three independent culturing sessions for bassoon colabeling assay; *N* =30 cells from three independent culturing sessions for PSD-95 colabeling assay. Note that the inability to phosphorylate Y142 residue following HGF treatment results in no change in the colabeling of presynaptic/postsynaptic markers. *p < 0.05, **p < 0.01 (HGF vs PBS).

Previous studies have demonstrated a role for an N-cadherin/β-catenin complex in synaptic vesicle localization at the synapse (Brigidi and Bamji, 2011). Given our data showing that, following HGF stimulation, there is a reduction of the MET/β-catenin complex that is accompanied by an increase in the β-catenin/N-cadherin complex, we wondered whether the HGF upregulation of Syn-GFP/bassoon and Syn-GFP/PSD-95 clusters requires dissociation of the MET/β-catenin complex. To address this, we transfected primary cultures of neocortical neurons with wild-type β-catenin or β-cateninY142F, which can form a complex with MET, but does not dissociate from the complex after HGF stimulation. Consistent with our data in untransfected cultures, HGF increased the density of Syn-GFP/bassoon and Syn-GFP/PSD-95 clusters in neurons transfected with wild-type β-catenin (Fig. 6*A*). In contrast, there was no increase in the density of these clusters in β-cateninY142F-transfected neurons (Fig. 6*B*). Quantitative analysis demonstrated a significant interaction between the construct transfected (wild-type β-catenin or β-cateninY142F) and treatment (PBS or HGF) on the density of Syn-GFP/bassoon (*F*_(1,92)_ = 8.056, *p* = 0.0056) and Syn-GFP/PSD-95 (*F*_(1,116)_ = 4.141, *p* = 0.0441) clusters. Pairwise analysis revealed that, in the absence of HGF, there was no significant difference in the density of Syn-GFP/bassoon (*p* ^v^ > 0.9999, df = 92; Fig. 6*C*) or Syn-GFP/PSD-95 (*p* ^y^ > 0.9999, df = 116; Fig. 6*D*) clusters in neurons transfected with wild-type β-catenin or β-cateninY142F. This result suggests that β-cateninY142F does not disrupt Syn-GFP/bassoon or Syn-GFP/PSD-95 cluster density under conditions in which MET is not stimulated. Following the addition of HGF, however, there was a significant increase in the density of Syn-GFP/bassoon (*p* ^w^ = 0.0232, df = 92) and Syn-GFP/PSD-95 (*p* ^z^ = 0.0018, df = 116) clusters in neurons transfected with wild-type β-catenin, but not in flag-β-cateninY142F-transfected neurons (Syn-GFP/bassoon: *p* ^x^ = 0.3078, df = 92; Syn-GFP/PSD-95: *p* ^aa^ > 0.9999, df = 116), compared with PBS. These results demonstrate that HGF activation of MET promotes an increased density of Syn-GFP/bassoon and Syn-GFP/PSD-95 clusters by regulating the MET/β-catenin complex through the phosphorylation of β-catenin at Y142.

## Discussion

Many genes have been identified as components of molecular networks involved in ASD risk (Bourgeron, 2009; Pinto et al., 2014). These putative relations are placed in a functional context by examining how interactions at the protein level impact typical and atypical neurodevelopment (Xie et al., 2016). In this context, we demonstrate here a dynamic, ligand-dependent functional interaction, either directly or indirectly, between two proteins encoded by ASD risk genes, *MET* (Campbell et al., 2006, 2008; Jackson et al., 2009; Thanseem et al., 2010; Zhou et al., 2011; Rudie et al., 2012; Abrahams et al., 2013; Lambert et al., 2014) and *β-catenin* (O’Roak et al., 2012a,b). A possible association between MET and WNT/β-catenin was recently put forth as a contributing mechanism through which neurodevelopmental events impacted in ASD are coordinated (Mullins et al., 2016). Previous reports of functional interactions between MET and β-catenin have focused on transcriptional regulation, in which HGF promotes the phosphorylation of β-catenin at Y142 directly via activating the MET receptor (David et al., 2008), followed by the dissociation of β-catenin from a MET complex and then translocated to the nucleus (Herynk et al., 2003; Rasola et al., 2007; Bhardwaj et al., 2013). We demonstrate a similar modulation of the MET/β-catenin functional interaction by HGF at the neocortical synapse. In contrast with nuclear translocation, however, the released β-catenin is, at least in part, sequestered into N-cadherin complexes, with activated MET receptor recruiting additional synapsin 1 to form functional complexes that may be mediated through other proteins in a complex. In addition to MET, phosphorylation of β-catenin at Y142 could also be induced by other non-receptor tyrosine kinases such as Fer or Fyn tyrosine kinase. This latter phosphorylation downregulates the interactions of β-catenin and α-catenin, but does not affect the β-catenin and cadherin adhesive complex, which is controlled by the phosphorylation of β-catenin at Y654 (Roura et al., 1999; Piedra et al., 2003; Tai et al., 2007). Independent of HGF/MET signaling, the regulation of cadherin/β-catenin/α-catenin complex by Fer and Fyn tyrosine kinases also contributes to synapse development (Bamji et al., 2006; Arikkath and Reichardt, 2008; Lee et al., 2008).

The dissociation of the MET/β-catenin complex is required for the HGF-induced increase in the density of Syn-GFP/bassoon and Syn-GFP/PSD-95 clusters in neocortical neurons. This increase, which is observed over a short assay period of 10 min, is suggestive of an increased number of nascent synapses in the presence of HGF and is reminiscent of increased synapse density, defined by synapsin 1 and PSD-95 colocalization, in the same culture paradigm after stimulation with HGF for 24 h (Eagleson et al., 2016). It should be noted that MET signaling also appears to modulate excitatory synapse maturation in the hippocampus and neocortex (Qiu et al., 2014; Peng et al., 2016), such that genetic deletion of *Met* results in premature maturation, assessed morphologically, electrophysiologically, and by the increased membrane insertion of AMPA receptor subunits (Qiu et al., 2014). Overexpression of MET in hippocampal neurons or slices *in vitro* results in both immature spine growth and electrophysiological properties. The data together are consistent with a unique dual role for MET during synapse development: initiating synapse formation at early stages and maintaining an immature functional state until MET signaling is eliminated. *In vivo*, MET receptor activation is robust during the period of rapid synapse formation between P7 and P14, but then rapidly falls to near-negligible levels by P16 (Eagleson et al., 2016), a time when neocortical synapses are undergoing maturation. While speculative, MET contribution to new synapse formation, followed by a decline to permit maturation, may contribute to the generation of an appropriate number or function of mature excitatory synapses in the developing neocortex. The disruption of MET signaling does increase excitatory drive and synapse maturation, and thus may alter excitation/inhibition balance, a key element contributing to neurodevelopmental disorders, including ASD (Rubenstein and Merzenich, 2003; Levitt et al., 2004; Dani et al., 2005; Gogolla et al., 2009; Gatto and Broadie, 2010; Bateup et al., 2013).

In the current study, we focused on the role of MET/β-catenin complex in modulating presynaptic development. Specifically, HGF increases synaptic vesicles clustering at the active zone and at the synapse through regulation of MET/β-catenin complex by the phosphorylation of β-catenin at Y142. We also showed that, under basal culture condition, β-catenin as well as β-catenin Y142F itself could promote synapse formation. This may reflect the role of β-catenin independent of HGF/MET signaling and phosphorylation of β-catenin at Y142 as discussed previously (Arikkath and Reichardt, 2008; Sun et al., 2009; Brigidi and Bamji, 2011), as well as the low concentration of HGF in our basal culture condition. The low concentration of HGF may not generate sufficient phosphorylation of β-catenin at Y142 to induce difference between β-catenin and β-cateninY142F-transfected neurons. It should be noted that the regulation of MET/β-catenin complex by HGF may occur at both presynaptic and postsynaptic sites, because *in vivo*, both β-catenin and MET are also localized at postsynaptic sites in the neocortex and hippocampus (Phillips et al., 2001; Murase et al., 2002; Eagleson et al., 2013). Thus, the increased alignment of synaptic vesicles at active zone and at synapse induced by HGF could be triggered through regulation of both presynaptic and postsynaptic MET/β-catenin complexes in the cultured neurons. It also should be noted that MET is localized predominantly in presynaptic compartments, and there is no individual synapse with MET distributed at both presynaptic and postsynaptic sites in the developing neocortex *in vivo* (Eagleson et al., 2013). While it awaits formal testing, we favor the hypothesis that the major regulation of MET/β-catenin complexes would occur at the presynaptic site in the developing neocortex *in vivo*. However, we note that β-catenin and MET have been demonstrated independently to modulate several features of postsynaptic development. For example, β-catenin regulates dendritic morphogenesis, dendritic spine density, and postsynaptic structure and function (Murase et al., 2002; Yu and Malenka, 2003, 2004; Abe et al., 2004; Gao et al., 2007; Okuda et al., 2007). Similarly, there is increasing evidence that MET signaling modulates dendritic and spine morphogenesis, as well as the clustering of postsynaptic proteins, including PSD-95 (Gutierrez et al., 2004; Lim and Walikonis, 2008; Judson et al., 2010, 2011; Finsterwald and Martin, 2011; Qiu et al., 2014; Peng et al., 2016). Thus, it is plausible that the MET/β-catenin complex, and the regulation of this complex by HGF, may influence different aspects of presynaptic and postsynaptic development.

The analyses of HGF-induced regulation of MET/β-catenin functional interactions in neocortical neurons *in vitro* raise the issue of defining the cellular and circuit context in which this signaling system may operate *in vivo*. At P14, we found that there are both colabeled and single-labeled neurons expressing *Met* and *β-catenin* transcripts in layers II-III and V-VI. The double-labeled neurons located superficially are almost entirely intrinsic intratelencephalic cortico-cortical or callosal neurons, whereas deep layer MET^+^ neurons could be both callosal and cortico-fugal in nature. During the active period of synaptogenesis in the mouse neocortex, *Hgf* mRNA is evident mostly in deep layers of the neocortex at P14 (Eagleson et al., 2016). Thus, both ligand and receptor are positioned to modulate MET/β-catenin complexes in subsets of neurons *in vivo*. Determining the specific subpopulation identities is currently under investigation.

Our current findings are consistent with converging evidence that the developing neocortical synapse is disrupted in ASD, with many ASD risk genes having implicated or demonstrated roles in synapse development and plasticity (Zoghbi, 2003; Garber, 2007; Südhof, 2008; Zoghbi and Bear, 2012; De Rubeis et al., 2014; Xie et al., 2016). Less progress has been made in understanding the heterogeneity in clinical presentation, which likely reflects the polygenic nature of the disorder. Our data suggest that an understanding of ASD risk at the level of protein functional interactions, including the identification of the specific subpopulations of neurons and circuits in which these interactions occur, will provide insight into how such heterogeneity arises. For example, MET expression in the primate brain is enriched in temporal, posterior parietal, and occipital regions, with very limited expression in a few frontal lobe areas. Neuroimaging studies confirm that the MET promoter risk variant impacts the structure and function of circuits in which it is enriched (Rudie et al., 2012). At the cellular level, the RNAscope analyses reveal that a subset of neocortical neurons coexpress MET and β-catenin during the peak period of synaptogenesis. This suggests that the biological impact of reducing MET expression, which occurs in ASD and Rett syndrome (Campbell et al., 2007; Voineagu et al., 2011; Plummer et al., 2013), may differentially disrupt the development of subpopulations of neurons, with specific changes being dependent on the specific repertoire of MET-interacting proteins expressed by different neurons and circuits. Advances in multiplex *in situ* techniques will provide opportunities to more carefully characterize the coexpression of multiple members of the MET interactome, 11% of which have been associated with neurodevelopmental disorders (Xie et al., 2016), in discrete neocortical neuron subpopulations.

## Synthesis

The decision was a result of the Reviewing Editor Robert Kalb and the peer reviewers coming together and discussing their recommendations until a consensus was reached. A fact-based synthesis statement explaining their decision and outlining what is needed to prepare a revision is listed below. The following reviewers agreed to reveal their identity: Randall Walikonis, Charles Finsterwald

Both reviewers find the topic interesting and novel. There is enthusiasm for the report. The two reviewers differ in their opinions about whether the work is appropriate controlled and cautiously interpreted. One of the key issues is localization and direct v. indirect protein-protein interactions. I think the authors can address this with new experiments and so I favor returning the ms. to them for revision and re-review

Dear Authors,

I have redacted portions of both reviewers comments and my synthesis is highlighted by ***. I think you will find my comments helpful as enthusiasm of the external reviewers for the revision will be largely determined by how well the highlighted issues are addressed.

1. The conclusions of this paper are heavily dependent upon the assumption that the proximity ligation assay shows direct interaction between proteins. However, signals from PLA simply mean that the target proteins are within about 40 nm of each other, and proteins in proximity to each other within a cellular subdomain or protein complex can give a PLA signal even if they are not directly interacting. The conclusions about the data that is shown must be tempered with this understanding.

***The authors could be more careful about their conclusion as for the direct interaction between Met and b-catenin or synapsin. Bringing new methods for addressing this critical issue would strongly help the cause. A yeast 2 hybrid system approach or GST-fusion protein approach are potential paths forward

2. MET and beta-catenin are probably also present at the postsynaptic compartment. Thus interpreting the data as if it was only in the presynaptic compartment is unfounded.

***I agree, the authors should be more careful about that interpretation. It is very important to be circumspect with interpretation.

Figure 1. Panels A-C. This is the best data showing a possible interaction between MET and beta-catenin, although it is possible that there are other proteins that are interposed between MET and beta-catenin. The blot against N-cadherin is an important piece of data, but additional control blots against other proteins could help eliminate the possibility of other intervening proteins. Postsynaptic proteins should also be included - even though the paper is written with a focus on presynaptic events, synaptosomes include postsynaptic complexes that can contain both MET and beta-catenin.

***Yes, additional pre and post synaptic proteins could be included as controls.

Panels B and C. The bands from multiple replicates should be quantified and graphed, particularly the N-cadherin (B) and beta-catenin (C) panels.

***I clearly agree with that comment.

Panel D. The staining for MET shows a very broad distribution, apparently along dendrites in these images. Is beta-catenin only found in the areas where the PLA gave a reaction? Are these synaptic sites where MET is also found? The PLA data could be showing postsynaptic proximity between MET and beta-catenin, so even though the manuscript is written from a presynaptic perspective the data doesn't necessarily result from presynaptic MET only. Parallel data showing double staining against beta-catenin with the same PLA conditions would be helpful. In the methods, the authors state that spines couldn't be seen based upon the imaging conditions. However, staining against a synaptic marker such as PSD-95 in a third channel to show the relative location of the PLA signal would be very informative.

***The authors must clarify MET and beta-catenin subcellular distribution. An in vivo characterization would be useful.

Figure 2. Panel A. This is the sole evidence presented that MET interacts with synapsin, although it doesn't eliminate the possibility that there are other intervening proteins linking MET with synapsin. Other synaptic vesicular proteins should also be stained against, and multiple replicates quantified. Other interaction assays between synapsin and MET should also be included, as this putative interaction forms a significant part of their model.

***Data should be quantified.

Panel B. The proximity ligation assay between MET and synapsin should be interpreted much more narrowly. It shows a close proximity of these two proteins, but not necessarily a direct interaction. In addition, if HGF induces synaptic activity (as has been shown previously), synapsin can translocate with vesicles to the active zone near where MET may be located, thus allowing for greater PLA signal that is independent of a direct interaction.

***Again the direct v. indirect issue is raised and needs better experiments to resolve the issue.

Figure 3. The western blot against pY142-beta-catenin and the number of clusters in neurons should be quantified.

***I agree.

2) the FLAG-beta-catenin signal appears to be dendritic (an image of a transfected whole neuron or a costain against an axonal or dendritic marker would be revealing), and its presynaptic function is thus unclear;

***Yes, a better discrimination of the pre vs. postsyanptic localization of b-catenin is essential

3) the apparent FLAG beta-catenin stain in dendrites is similar to the MET stain in dendrites (Figure 1), so why is there no PLA signal throughout the dendrite? Are they only colocalized at synaptic sites?

***This is a very good point raised. In general, I would also suggest to present all PLA data with the 2 channels used.

4) This Y142 phosphorylation site regulates the switch for beta-catenin between its adhesive role and its transcriptional role. Wouldn't the Y142F mutant thus cause beta-catenin to remain in an adhesive state with alpha-catenin and thus remain at synapses, independent of a putative interaction with MET? Similarly, phosphorylation of WT beta-catenin would cause it to be released from its interaction with alpha-catenin and thus translocate away from sites near MET.

***Separation between phospho-b-catenin and MET could indeed be mediated by its interaction with alpha-catenin. This however does not change the fact that HGF treatment leads to b-catenin phosphorylation and separation from MET. The authors could discuss this interesting point.

5) Given the limitation of the PLA in showing direct interactions, it would be more convincing if a Co-IP was conducted similar to figure 1 against endogenous proteins showing a loss of interaction upon phosphorylation of beta-catenin. At minimum, the FLAG-beta-catenin should be shown to become phosphorylated upon HGF treatment.

***A control showing that exogenous flag-b-catenin becomes phosphorylated upon HGF stimulation should be added.

Figure 5. Why is GFP-synapsin used instead of simply staining against endogenous synapsin? This would eliminate artifacts due to long transfection times and overexpression artifacts. The data on the size of the Syn-GFP clusters suggests that the clusters get larger with treatment with HGF. Larger clusters, simply due to their increased size, will overlap to a greater extent with the abundant puncta of other synaptic markers.

***Endogenous staining of synapsin would be more physiological than Synapsin-GFP expression.

Figure 6. This data would be more convincing if done with antibodies against endogenous synapsin. The Syn-GFP forms very large clusters that may or may not mimic endogenous events. It is not clear from the images whether the Syn-GFP and FLAG-beta-catenin are in dendrites or axons. An image of an entire transfected neuron or showing a costain against a dendritic or axonal marker would clarify. The caveat about overexpressing these proteins for an extended amount of time is also applicable to this experiment.

***Endogenous staining of synapsin would be more physiological.

Sincerely,

Robert Kalb, MD
